# Automated Bowel Sound Analysis: An Overview

**DOI:** 10.3390/s21165294

**Published:** 2021-08-05

**Authors:** Jan Krzysztof Nowak, Robert Nowak, Kacper Radzikowski, Ireneusz Grulkowski, Jaroslaw Walkowiak

**Affiliations:** 1 Department of Pediatric Gastroenterology and Metabolic Diseases, Poznan University of Medical Sciences, 60-572 Poznan, Poland; jarwalk@ump.edu.pl; 2 Artificial Intelligence Division, Institute of Computer Science, Warsaw University of Technology, 00-665 Warsaw, Poland; robert.nowak@pw.edu.pl (R.N.); kradziko@mion.elka.pw.edu.pl (K.R.); 3 Faculty of Physics, Astronomy and Informatics, Institute of Physics, Nicolaus Copernicus University, 87-100 Toruń, Poland; igrulkowski@fizyka.umk.pl

**Keywords:** bowel sound, automated analysis, intestine, motility, recording

## Abstract

Despite technological progress, we lack a consensus on the method of conducting automated bowel sound (BS) analysis and, consequently, BS tools have not become available to doctors. We aimed to briefly review the literature on BS recording and analysis, with an emphasis on the broad range of analytical approaches. Scientific journals and conference materials were researched with a specific set of terms (Scopus, MEDLINE, IEEE) to find reports on BS. The research articles identified were analyzed in the context of main research directions at a number of centers globally. Automated BS analysis methods were already well developed by the early 2000s. Accuracy of 90% and higher had been achieved with various analytical approaches, including wavelet transformations, multi-layer perceptrons, independent component analysis and autoregressive-moving-average models. Clinical research on BS has exposed their important potential in the non-invasive diagnosis of irritable bowel syndrome, in surgery, and for the investigation of gastrointestinal motility. The most recent advances are linked to the application of artificial intelligence and the development of dedicated BS devices. BS research is technologically mature, but lacks uniform methodology, an international forum for discussion and an open platform for data exchange. A common ground is needed as a starting point. The next key development will be the release of freely available benchmark datasets with labels confirmed by human experts.

## 1. Introduction

Out of nearly eight billion people currently inhabiting the Earth it is likely that almost all emit and have heard bowel sounds (BS). Yet, very few are able to exploit these BS for patients’ benefit. BS are an absolutely ubiquitous phenomenon and—as a highly intuitive subject of study—have attracted attention since antiquity, leading to discoveries of altered BS in diseases ranging from ileus to diarrhea [1]. As BS are generated by contractions of the intestine [2], they naturally reflect its motoric activity, which is notoriously difficult to measure directly (invasively) and assess [3].

BS can be compared to heart sounds, which have proved fundamental for clinical medicine, as they are (1) closely linked to vital processes underlying life and health, (2) produced frequently (albeit not rhythmically), (3) affected by a broad range of intrinsic and extrinsic factors. BS can therefore be considered a vital sign, especially when bowel function is lost or severely disturbed. Despite this evidence of the usefulness of BS, their use has been hampered by a lack of effective tools, and even negative opinion largely stemming from the limits of brief clinical auscultation [4,5].

BS are challenging to investigate because of their seemingly random nature, extremely broad dynamic range, and potential dietary influence. As we demonstrate in this review, modern technologies circumvent some of these limitations. Automated identification of bowel sounds already enables their use as a vital sign [6]. Moreover, it seems that extraction and analysis of more complex data (patterns, variability, spectra) may uncover in more detail the basic physiology of the gastrointestinal tract and the nature of its maladies.

Despite technological progress, practical BS solutions are not available to doctors. We are therefore at a turning point when technological BS analysis maturity is being translated into practice and, hopefully, will propel clinical BS research to unravel its true potential. To facilitate this transition, we aimed to briefly review the literature on BS recording and analysis. We show the current position of BS research and attempt to diagnose the missing elements that currently thwart the widespread application of automated BS analysis in clinical practice.

## 2. Materials and Methods

On 19 July 2018 and then on 11 March 2021 Scopus (Elsevier, Amsterdam, The Netherlands) was queried with the following term: TITLE(“abdominal sound*”) OR TITLE(“bowel sound*”) OR TITLE(“intestinal sound*”) OR TITLE(“bowel noise*”) OR TITLE(“abdominal noise*”) OR TITLE(phonoenterography) OR KEY(“abdominal sound*”) OR KEY(“bowel sound*”) OR KEY(“intestinal sound*”) OR KEY(“bowel noise*”) OR KEY(“abdominal noise*”) OR KEY(phonoenterography). The search yielded 218 documents. The abstracts, if available, were reviewed. One article known to the authors and retrieved by a similar search on MEDLINE was added to the list [7], and two other articles were identified from other sources [8,9]. We searched MEDLINE and IEEE.

## 3. Results

### 3.1. 1950s–1960s

**Figure 1:**
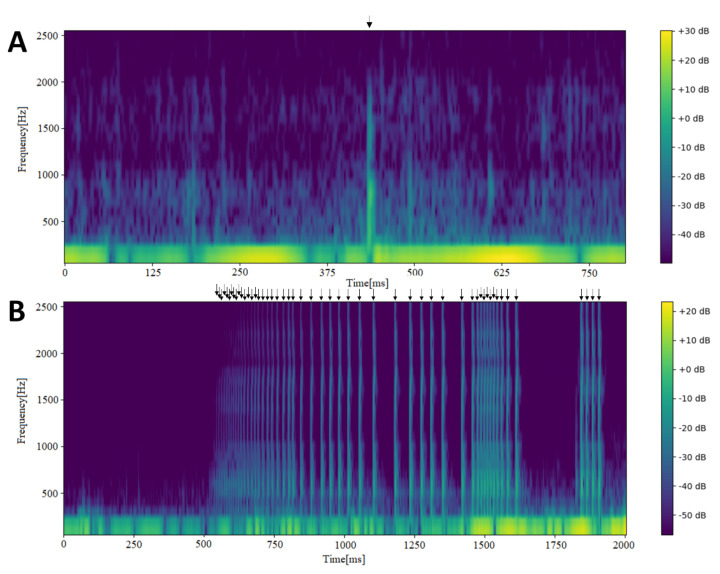
Spectrograms depicting bowel sounds recorded using a contact microphone in the right lower quadrant of the abdomen. (**A**) An individual short bowel sound is clearly discernible in the middle (~425 ms; indicated by the arrow at the top). The signal below 250 Hz is composed of venous hum and heart beats. (**B**) A series of over 50 bowel sounds (arrows) occurring in less than 1.5 s.

One of the earliest articles on the link between BS (Figure 1) and intestinal motility was published in 1955 [10]. In 1958, the absence of BS in severely ill patients was described [11]. Sprung and Roisch first mentioned the recording of BS for the purpose of small intestinal motility assessment [12,13]. Two years later, the first intestinal stimulation test with BS analysis was performed [14]. In the early 1960s, BS were the subject of a work by Von Ardenne et al. [15]. Five years later, apparatuses for the registration of BS were presented by two teams [16,17]; in 1967 the term “phonoenterography” was introduced in an article published in “The Gut” [18].

### 3.2. 1970s

The 1970s saw the development of new themes in BS research: computer [19] and frequency [20] analysis, pre- and postprandial BS recording [21], and the search for the anatomical origin of BS [22]. The first approach to computerized analysis involved a lower threshold for sound amplitude and an upper threshold for its duration [19]. The typical frequency of abdominal sounds was found to be either 100 or 300 Hz, although this also included stomach rumblings.

### 3.3. 1980s

The 1980s brought a still better understanding of BS. It was found that in neonates, BS rarely exceed 1500 Hz [23]. The usefulness of the phonoenterogram was proposed in the diagnosis of acute ileus [24], kidney failure [25], and acute appendicitis [26]—all of which produce acute abdomen with reduced peristalsis. Further studies on pre- vs. postprandial BS were conducted in the frequency domain [27]. Phonoenterography with diurnal BS variation analysis was found to be of use in pediatrics [28]. In a proof-of-concept study the use of BS biofeedback with an electronic stethoscope was found to have considerable therapeutic potential in irritable bowel syndrome [29,30].

Five-channel BS analysis was performed using dedicated equipment (Phonoenteroanalyzer PEA-06) with purpose-built microphones [31]. Signal processing and analysis involved rectifying, peak detection and logarithmic transformation. Artifacts resulting from movements were controlled using an additional microphone and breathing analysis. Phonoenteroanalyzer PEA-06 was used for overnight recordings and during three different stimulation tests. The authors stressed that the method was “objective, continuous, non-invasive”.

### 3.4. 1990s

In the 1990s, research on BS analysis accelerated (Figure 1). Yoshino et al. distinguished three types of BS with peak frequencies of 273, 632 and 612 Hz. The different types of sound were associated with the need for surgical treatment [32]. Long-term, real-time BS analysis using a microprocessor gave encouraging results, despite external and internal noise [33]. The application of a computer-aided sound analysis system using 124 people in a silent environment over 10 min periods revealed that the mean BS length was 20 ± 1 ms in healthy people and could increase more than twofold in acute surgical pathology, such as cholecystitis or intestinal obstruction [34]. The number of BS and their amplitude were also affected by diseases. The system was proposed for use mainly in surgical wards, but also for pharmacological studies and motility disorder diagnostics [35]. Other apparatus for automated BS analysis was introduced by a Chinese group [36]. In addition, BS were automatically analyzed in a rat model of small bowel obstruction [37] by a team which moved on to create a signal filtering solution to remove noise from BS recordings [38]. The team used signal envelope (Hilbert transform) and amplitude thresholding with good results [39]. Finally, in 1999, Craine et al. reported large differences in sound-to-sound intervals between patients with irritable bowel syndrome and healthy controls (452 ± 35 ms vs. 1931 ± 365 ms, *p* = 0.0001) [40]. The cut-off value was set at 640 ms, yielding a sensitivity of 91% and specificity of 100%. Their work remains one of the most impressive examples of the clinical potential of BS analysis.

### 3.5. Thessaloniki Group

Concurrently, in 1999, Hadjileontiadis et al. of Aristotle University of Thessaloniki in Greece applied a multiresolution analysis with hard thresholding (wavelet transform-based stationary–nonstationary filter) to BS, which were further analyzed using higher-order crossings [41]. The authors reported excellent results with no need for a reference microphone. In their view, the method was ready for clinical use [42]. The team later proposed kurtosis-based [43] and fractal dimension analysis of BS (and lung sounds), which was robust to changes in duration and in amplitude [44,45]. Their final work focused on wavelet-based algorithms with fractal dimension analysis [45,46,47]. Hadjileontiadis argued that a wavelet transform-based stationary–nonstationary filter could be applied in real-time due to the low computational cost. Further BS projects conducted in Thessaloniki by Dimoulas et al. concerned Wiener filtering with discrete wavelet transform and wavelet packets [48]. Dimoulas et al. produced a system for BS segmentation, visualization and browsing [49]. They also proposed the use of neural networks in BS analysis [50], in order to permit long-term BS recording and motility disorder diagnosis. The model involved time–frequency, wavelet parameters and multi-layer perceptrons. The accuracy of recognition was 95%. The authors also developed other solutions for long-term BS recording and automated analysis, including a dedicated visual-assisted application [51]. The software, intended for long-term multichannel BS recordings, was further improved in 2016, notably offering sound localization capabilities [52].

### 3.6. Nancy Group

In 2001, Ranta et al. presented unsupervised denoising, segmentation and characterization of BS. They optimized the algorithm by Hadjileontiadis et al. and recorded patients [53]. In the following decade this group continued BS research to analyze the anatomical distribution of BS and propose a source localization method [54], perform principal component analysis of BS [55] and obtain more in vivo recordings [56]. The habilitation thesis of one coauthor, V. Louis-Dorr, contains details of the work and can be found in the French open archives [57]. The work of Ranta et al. is particularly interesting in the context of multichannel recording and spatiotemporal BS distribution [56].

### 3.7. Yamanashi/Tokyo Group

In 2004, Sakata et al. used high-sensitivity accelerometers in place of microphones, along with an independent component analysis algorithm, to automatically detect BS [58]. They then added a wavelet filter to their scheme, improving the overall results; a detection ratio (sensitivity) of 99% with <2% false positives was achieved [59]. Sakata et al. also showed that BS recordings are a source of patients’ stress [60] and that lifestyle changes may influence BS more than types of food [61]. They also demonstrated that the frequency of low and normal intensity BS did only correlate to periods of digestion [62]. In recent work led by Sakata, a bowel sound monitoring system was employed to better understand the relationship between motility and inflammation, indicated by interleukin-6 concentration in the blood [63]. Together with Yamada, Sakata also described a two-step approach to real-time BS identification under noisy conditions, for use in intensive care units [64]. Sakata’s group was successful in predicting BS occurrence using the seasonal autoregressive integrated moving average (SARIMA) model [65], a feat that paves way for identifying new intestinal physiology. Kodani and Sakata attempted to solve the problem of clothes rubbing against the microphone, which produces artifacts that practically preclude any bowel sound analysis in a moving subject. Application of a number of filters (notch, wavelet, low-pass) was successful [66]. Although the presence of many loud artifacts is likely to decrease the number of high-quality recordings available for analysis, considerable diagnostic value may be retained even with intermittent high-quality sampling. This stresses the need for microphones specific for BS, a challenge that was also addressed by this group by joining microphones and a vibration sensor [67].

### 3.8. Jeonju Group

Interesting work on BS analysis was done by Kim et al. who conducted a regression analysis of BS shimmer and jitter. They demonstrated a strict correlation with radiographic colon transit time in a preliminary study [68] and proposed a back-propagation neural network model of BS [69]. Kim et al. underscored the potential of the method to non-invasively monitor bowel motility [70]. Interestingly, Kim et al. mention recording the ascending, transverse and descending colons. It is known that various segments of the colon are superimposed—the abdomen is heterogeneous and therefore sound localization constitutes a challenge [56].

### 3.9. Tokushima Group

In 2013, Emoto et al. made an observation that remains pertinent today: although BS recording is non-invasive and inexpensive, there is no consensus regarding automated analysis protocol [71]. They proposed an algorithm that was 88% sensitive and 92% specific—a 3 dB bandwidth of “frequency peaks in the autoregressive moving average spectrum”. The indicator selected by Emoto et al. as the most relevant was the sound-to-sound interval. We fully concur with the Japanese team that a general agreement on the method should be reached and that it should build on the simplest solution available to allow for more knowledge on clinical relevance to be gathered. Emoto et al. also studied BS with non-contact microphones [72]. The team achieved automated BS identification through an autoregressive moving average of 91% accuracy and concluded that BS duration reflects intestinal motility [73]. The group successfully applied unsupervised BS detection in non-contact recordings and determined three clinically pertinent parameters: sound-to-sound interval, which was associated with frequency (per minute), as well as the ratio of signal to noise [74]. These achievements can be considered a successful application of artificial intelligence-based BS detection in non-contact recordings.

### 3.10. Los Angeles Group

In 2014, Spiegel et al. designed a disposable, non-invasive acoustic biosensor to monitor bowel sounds across the acoustic spectrum, including hertz ranges [8]. This acoustic gastrointestinal surveillance system included software counting acoustic motility events based on frequency and reporting the number of their occurrences per minute—a metric called the ‘intestinal rate’. This setup was used in 28 postoperative patients to successfully detect postoperative ileus [9]. The acoustic gastrointestinal surveillance system achieved approximately 80% sensitivity and specificity, 83% area under the ROC curve, and showed its usefulness in advancing patients’ diets. Crucially, this system has obtained regulatory approval from the American Food and Drug Administration (FDA).

### 3.11. Perth Group—The Noisy Guts Project

Many of the recent advances have been spearheaded by an Australian team led by Prof. Barry Marshall. In 2018, Inderjeeth et al. performed a systematic review of BS analysis methods and concluded that none of the 14 selected approaches were ready for clinical application [75]. In the article that followed, they demonstrated the capability of a low-cost system employing piezoelectric transducers as contact microphones to identify migrating motor complexes [76]. We were already testing a prototype piezoelectric BS microphone when the article was published and can confirm that this method is promising. However, the Australian team has accomplished this with refined machine learning approaches that are now freely available and which were reviewed methodologically by The Noisy Guts team in 2019 [6]. Moreover, the group proposed a mathematical model of BS generation [77], and a clinical application of the completed BS framework soon followed. The results of their proof-of-concept investigation of BS analysis for irritable bowel syndrome diagnosis are inspiring: 87% sensitivity and specificity were achieved in an independent cohort [78]. It would seem that the Australian team is heading towards the production of a medical device for BS recording and analysis. Overall, we consider the systematic and multifaceted work of The Noisy Gut Project as a turning point in BS research. Inderjeeth et al. are positive about BS analysis value generally, probably due to their two studies indicating excellent future potential. We consider this optimism is much needed in a field that has seen a number of proof-of-concept demonstrations but too few practical applications.

### 3.12. Other Recent Technological Developments

The 2000s brought about other technological developments from various groups. In 2002, a letter by a Japanese team elaborated on the theme of automated BS analysis [79]. Jeon et al. used an electronic stethoscope and WAV (wave form audio format) file analysis to study the relationship between meal ingestion, 5-hydroxytryptamine concentration in the blood and BS [80]. A computer phonoenterography study by a Russian team assessed BS of healthy children in the context of fasting and the first meal of the day [81].

In 2008, a system for the recording of BS in preterm neonates was proposed [82]. The same year, a Chinese team reported on a peripheral circuit with USB (Universal Serial Bus) controller for real-time BS “detection, display and storage” [83]. Zhang et al. also reviewed BS detection algorithms (in Chinese) [84]. In 2009, a device was developed that allowed visualization of BS intensity over the abdomen in the form of a colored map [85]. It is clear that such a device could prove valuable for pre- or post-surgical monitoring. Additionally in 2009, Delfini et al. used five electret microphones attached to a PC to acquire signals from five volunteers and further analyzed them in LabVIEW and MatLab [86]. In 2011, Tsai et al. recorded BS with an electronic stethoscope placed in the right lower abdominal quadrant, and applied LabVIEW filtering using digital infinite impulse responses to automatically analyze the signal [87]. A more recent study using LabVIEW classified BS into absent, hypo- and hyperactive [88].

Li et al. automatically extracted BS by assessing their energy and duration; afterwards adaptive noise filtering was applied and characteristics were calculated [89]. Lin et al. used a higher order statistical approach to amplify BS [90]. In 2014, Ulusar presented methodology for real-time and long-term BS monitoring, based on a naïve Bayesian classifier, minimum statistics and spectral subtraction [91]. A ready-to-use portable BS monitoring apparatus was presented by Al Mamun et al., consisting of amplifiers, converters and signal processors, capable of demonstrating BS occurrence [92]. The system is also intended to inform the artificial pancreas [93], a challenge more recently addressed by using Mel-frequency capstral coefficients and wavelet entropy [94] and support vector machines [95]. Sheu et al. showed the efficiency of a higher-order-statistics fractal dimension method for the detection of BS in different types of noise [96]. Zaborski et al. employed adjustable grids to indicate differences between sounds recorded in patients with peritonitis and healthy volunteers [7].

A sensor system with two microphones—called the “ZigBee” module—and a wireless connection were prepared for real-time monitoring of BS after surgery [97,98]. Baronetto et al. from Erlangen developed an elastane T-shirt fitted with eight microphones, of which seven are located above the abdomen. This was used to obtain a signal in which over 3000 BS were manually annotated. Clustering revealed the presence of four main morphologies of BS [99]. Using an array of microphones maximizes the available information and may enable the tracing of space-related effects; this could potentially prove useful in characterizing migrating motor complexes.

In 2016, Wang et al. described a wearable BS recorder with multiple channels [100]. The same year, Zhou et al. mooted the possibility of detecting BS with spectral entropy analysis [101]. Huang et al. used time series Gaussian Hamming distance to identify BS of one type only [102]. What warrants attention here is the care the team took to simplify the solution, which finally yielded convincing results. Yin et al. used an artificial neural network for BS analysis after denoising with a least mean square approach. Domains of both time and frequency were analyzed by creating feature vectors [103]. The same team then experimented with Legendre fitting, obtaining positive results [104]. In 2018, the group, based in Beijing and Shenzhen, presented a wearable BS monitoring system making use of a support vector machine [105]. In 2018, a team from Tsinghua University in Peking demonstrated that voice recognition neural networks can be applied to BS with high accuracy [106]. A flexible, skin-mounted electronic device/sensor with wireless communication capacity was developed at the same university, intended for BS recording [107]. The Tsinghua University group used their wireless sensor to obtain 20 clinical recordings lasting 24 h, from which 8000 sound segments were extracted in order to train convolutional neural networks. The resulting accuracy of 92% demonstrated the high potential of practical application and the technology’s maturity [108].

In terms of analytics, Kölle et al. devised an intrinsic mode function-fractal dimension method for filtering artifacts, including the frequency range where BS typically occur [109]. Chen and Montlouis explored individual wave components as a basic building block for synthesizing artificial bowel sounds in the absence of large publicly available datasets, and confirmed its utility in extracting information by comparing it with a clinical sample [110]. This work, together with new solutions from the group in Tokyo, has contributed to differentiating between noise and BS.

Examples of various analytic techniques employed to study BS around the world are presented in Table 1.

### 3.13. Recent Clinical and Translational Progress

The progress of clinical research on BS has been relatively slow. A phonoenterography study conducted on 75 surgical patients confirmed the changes to BS in peritonitis [111]. A blinded study of doctors showed that clinical auscultation may be sufficient to detect ileus [112]. However, auscultation by qualified nurses was insufficient to guide feeding in critically ill patients [113] or detect ileus, according to a systematic review [114]. Using automated BS analysis, Ozawa et al. found that peristalsis is less active in Parkinson’s disease and multiple system atrophy, two important neurological diseases with possible gastrointestinal involvement [115].

In 2012, Ching et al. employed a commercially available electronic stethoscope to perform six 8 s recordings, replicating/reproducing a careful clinical examination [116]. The study was performed with 71 patients and involved basic time- and frequency-domain parameters. The auscultation was found to be insufficient for the diagnosis of intestinal obstruction, but useful for determining the possible site of obstruction. Felder et al. demonstrated weak predictive value of clinical auscultation for the diagnosis of small bowel obstruction and similar conclusions were reached by Brum et al. [5,117]. Li et al. reviewed the literature on the utility of BS in intensive care units and concluded that the potential of their use was high, but reasonable practice was needed [118,119]. A randomized study questioned the utility of abdominal auscultation in surgery to indicate the end of postoperative ileus; this study, however, assumed that first flatus after surgery more accurately reflects motility than does acoustic bowel activity [120]. Another study showed no relevance of clinically assessed BS or flatus for the tolerance of feeding reintroduced after surgery [121]. On the other hand, Ulusar et al. presented a system that was able to analyze BS in real time in order to facilitate early feeding after surgery [122]. Struble and Moser stressed the variability of the sounds and their limited usefulness in diagnosing short bowel obstruction [123].

The insufficient reliability of clinical auscultation prompted Dumas et al. to construct a system for continuous BS monitoring in neonates; a single stethoscope was used, attached with a hydrogel patch for over 3 h. The authors suggested that a BS vital sign is needed and that a real-time analysis could help guide automatic feeding pumps [124]. BS recorded with a single stethoscope were used to investigate the efficacy of exercises on bowel motility [125,126]. Kusainov and Makukha demonstrated that a microelectromechanical system microphone may be used in an electronic stethoscope [127]. Of interest is Liu et al.’s demonstration that oscillating gas bubbles are the source of BS [2].

In 2018, Mohnani and Eisenfeld applied wavelet Bayesian denoising to demonstrate the capacity for continuous bowel sound monitoring in patients with necrotizing enterocolitis [128]. A score between 0 and 9 was used to summarize the level of BS activity. A 2018 study by Ortigoza et al. demonstrated the progress made in BS analysis up till then: it was combined with electroencephalography and near-infrared spectroscopy in a group of neonates to objectivize the assessment of the maturity of the neonates’ gastrointestinal tract [129].

In 2020, Wang et al. (Beijing) successfully applied denoising and wavelet decomposition to reveal anesthesia-related attenuation of intestinal acoustic activity [130]. Worth noting, the team reported on tests of acoustic parameters [131], which may be important for harmonizing various approaches in the future.

Overall, the timeline of BS clinical research demonstrates a struggle against technical difficulties, examples of physiologically informative BS analyses and a growing awareness of the limits of standard clinical auscultation.

## 4. Discussion

Automated BS analysis is an intuitive research subject with major potential for clinical application. Significant progress in relevant technical capability has been made since the 1990s globally, with a surge in the past decade, as evidenced by a series of studies reporting high accuracies (Table 2) and independently developed approaches to BS recording. The new frontiers are extraction of complex traits, optimization of recording protocols and techniques, artifact filtering, development of easily accessible software and hardware, harmonization of BS definitions, and the establishment of platforms for BS scientific debate, along with benchmark datasets.

Apart from technical aspects, further challenges in BS research need to be addressed by clinicians. First, the search for a relationship between the motoric activity of the gut and intestinal sounds needs to be described in more detail. Preliminary data suggests that antroduodenal and colonic manometry may contribute to this issue. Other motility studies and devices, such as oro-anal transit, wireless motility capsules, and even electrogastrography, might be helpful as well. These clinical methods may, however, prove insufficient to elucidate the origin of BS. Experimental studies will be needed to address this issue by generating models to describe relationships between luminal contents (gas, liquids, solids), contractions (strength, duration, propagation, distance), the intestine (orientation/shape, diameter, elasticity, surface irregularities), and the abdominal cavity (dimensions, pressure and its changes during respiration). It is likely that subtypes of BS exist because of differences in such properties. Physiology research on the influence of vagal tone, foodstuffs, inflammation and similar topics will also be important. There is a surprising lack of such experimental and physiologic studies. Firm establishment of mechanistic foundation for clinical BS research appears necessary.

Second, we need to determine BS reference values across different age groups, genders, dietary patterns, nutritional statuses, lifestyles and ethnicities. These studies will need to have not only cross-sectional, but also cohort design. It is possible that other factors, yet unknown, will show consistent relationships with BS parameters. These may involve medication, e.g., opioids or iron that cause constipation, as well as laxatives. Any future clinical application will probably very much depend on such knowledge.

Third, observational studies of patients with various diseases involving hypo- and hypermotility need to be conducted. Typical examples include irritable bowel syndrome—which was recently linked to previously overlooked food sensitization—and post-operative ileus. BS in diabetes have also been explored, but to inform the artificial pancreas algorithms; it is noteworthy that BS in diabetes may be disturbed because of diabetes-related disorders of small intestinal motility. Such problems could be encountered in scleroderma. An obvious candidate for BS characterization is chronic constipation, along with Hirschsprung’s disease. Other gastroenterological conditions include pancreatitis and cystic fibrosis, as well as enteral/parenteral feeding and infectious diarrhea. BS could also be explored in obesity (gastrointestinal hormones), cardiovascular disease, oncology (treatment toxicity), neurology (Parkinson’s disease, multiple sclerosis), nephrology, intensive care, thyroid diseases, and psychiatry. The possibilities are numerous, and they will likely be fully researched in the decade after clinicians receive a fully operational, reliable system that automatically provides quantitative data. Due to the non-invasive nature of the measurements it is possible that pediatric research will progress as quickly as that into adults, not always the case previously.

Fourth, the relationships between markers of intestinal homeostasis or function and BS need to be studied. Examples include fecal calprotectin, zonulin, chromatographic assessment of intestinal permeability, digestion and absorption of specific foodstuffs. A number of omics could be integrated into BS research, and methods could be adapted to better study animal models of gastrointestinal diseases.

The areas of research into BS continue to develop and broaden. However, the BS community first needs to bring into final fruition the very extensive work of several dedicated teams and dozens of individual researchers striving in this field to date.

## 5. Conclusions

Multiple diverse methods have proved to be efficacious in automated BS identification. Nevertheless, further progress is being hampered by a lack of standard methodology for use in a clinical setting. This could be resolved by providing clinicians with easily accessible equipment and analysis tools, linked to an international forum for discussion, and an open platform for data exchange. In accord with the intuition of Emoto and Huang, we propose that a simple solution be temporarily accepted as an international common ground and starting point, namely the occurrence of BS.

In our opinion, the issue of greatest immediate importance in automated BS research is the availability of a benchmark dataset containing high-quality recordings of bowel sounds with labels confirmed by human experts. Such a dataset could be used to compare different signal processing and classification methods. Our team is currently collecting such data and we plan to make our dataset freely available. Ideally, a bowel-sound centered platform would enable the sharing of other datasets, as well as comparison of different sensors and hardware systems for data collection.

We also call for the organization of a virtual meeting dedicated to BS, which could be undertaken by one of the teams with an excellent track record.

## Figures and Tables

**Table 1:** Diversity of analytic techniques enabling or facilitating the identification of bowel sounds (BS).

Group Home City	Examples of BS Research Techniques	
Antalya, Turkey	Naïve Bayesian classifier, minimum statistics and spectral subtraction	[91]
Beijing, China (multiple groups)	Convolutional neural networks, Legendre fitting, support vector machines, wavelet decomposition	[103,104,105,106]
Chengdu, China	Spectral entropy analysis	[101]
Jeonju, Korea	Regression analysis of BS shimmer and jitter, back-propagation neural network	[68,69,70]
Los Angeles, CA, USA	Bayesian classification, frequency-based counting	[8,9]
Nancy, France	Unsupervised denoising	[53,54,55,56]
Perth, Australia	Neural network: logistic regression–based machine learning	[76,77,78]
Saloniki, Greece	Wavelet transform-based stationary-nonstationary filter, higher-order crossings, kurtosis-based and fractal dimension analysis, neural networks	[41,42,43,44,45,46,47,48,49,50,51,52]
Singapore	Gaussian Hamming distance	[102]
Szczecin, Poland	Adjustable grids	[7]
Tainan, Taiwan	Higher-order-statistics fractal dimension	[96]
Tokushima, Japan	Autoregressive moving average spectrum	[71,72,73,74]
Tokyo, Japan	Independent component analysis with wavelet filtering, seasonal autoregressive integrated moving average	[58,59,60,61,62,63,64,65,66,67]
Trondheim, Norway	Intrinsic mode function-fractal dimension	[109]

**Table 2:** Accuracy of bowel sound identification methods developed by selected groups from around the world, demonstrating successful application of diverse approaches. However, accuracy reported by teams based in the cities listed cannot be directly compared because of largely different recording techniques, datasets and definitions of bowel sounds.

Group Home City	Accuracy	Years (Publications)
Saloniki, Greece	95%	1999–2011
Tokushima, Japan	91%	2013–2018
Antalya, Turkey	94%	2014
California, USA	83%	2014–2016
Beijing, China	92%	2018–2019
Perth, Australia	87%	2018–2020
Trondheim, Norway	75%	2019

## Data Availability

The review was based upon publicly available academic literature databases.
